# Qigesan inhibits esophageal cancer cell invasion and migration by inhibiting Gas6/Axl-induced epithelial-mesenchymal transition

**DOI:** 10.18632/aging.103238

**Published:** 2020-05-20

**Authors:** Lingyu Kong, Xin Lu, Xuanyu Chen, Yunyan Wu, Yushuang Zhang, Huijuan Shi, Jing Li

**Affiliations:** 1 College of Integrated Chinese and Western Medicine, Hebei Medical University, Shijiazhuang 050017, Hebei, China; 2 Department of Clinical Laboratory, Tangshan Maternal and Children Hospital, Tangshan 063000, Hebei, China; 3 Research Center, The Fourth Hospital of Hebei Medical University, Shijiazhuang 050011, Hebei, China; 4 Department of Traditional Chinese Medicine, Tumor Hospital of Hebei Province, The Fourth Hospital of Hebei Medical University, Shijiazhuang 050011, Hebei, China

**Keywords:** TCM formulas, ESCC, Gas6/Axl, epithelial-mesenchymal transition, cancer metastasis

## Abstract

Qigesan (QGS) has been used to effectively treat esophageal cancer (EC) for decades in China, but the mechanism by which it suppresses EC metastasis remains unknown. In this study, we examined the effects of QGS on EC cell mobility. Using immunohistochemistry and immunofluorescence, expression of Gas6 and Axl, which promote tumor cell migration and invasion, was examined in carcinoma tissues and adjacent normal tissues from EC patients. Levels of Gas6, Axl, and the Gas6/Axl complex were also examined in ECA109 and TE13 EC cells treated with QGS. In addition, immunofluorescent staining and quantitative protein analysis were used to examine E-cadherin, N-cadherin, and Snail levels in ECA109 and TE13 EC cells after QSG administration, and cell mobility was assessed. The results demonstrated that levels of Gas6 and Axl expression are higher in EC tissues than in adjacent normal tissues. Moreover, QGS decreased Gas6/Axl levels, increased E-cadherin expression, decreased Snail and N-cadherin expression, and inhibited epithelial-mesenchymal transition (EMT) in EC cells. QGS thus suppresses EMT in EC by inhibiting Gas6/Axl binding.

## INTRODUCTION

According to the 2018 global cancer statistics report, esophageal carcinoma (EC) is the seventh and sixth leading cause of worldwide cancer-related morbidity and mortality, respectively [1]. EC death rates remain high primarily because of tumor lymphatic metastasis and hematogenous dissemination [2]. EC is typically categorized as one of two pathological types; esophageal squamous cell carcinoma (ESCC) accounts for 80% of cases, while the rest are esophageal adenocarcinoma (EAC) [3]. EC is particularly common in Asia and southern Africa. In China, where EC incidence and mortality rates are highest, the incidence rate is 10 times higher than the world average, and ESCC is the most common pathological type [4, 5]. According to the Global Cancer Survival Trend Statistics Report 2010-14 (CONCORD-3), the 5-year survival rate of EC patients in China is lower than in neighboring Japan and South Korea [6]. In addition, most ESCC patients have advanced-stage disease at the time of diagnosis. Despite continuous advances in surgery and radiochemotherapy, tumor metastasis and recurrence, which are the primary causes of death in ESCC patients, remain relatively common [7–9]. However, the mechanisms underlying ESCC cell migration and invasion, the main causes of recurrence and metastasis, remain unclear, and additional studies are needed to examine the mechanisms underlying ESCC mobility.

Studies have shown that Gas6 and Axl are highly expressed in hepatocellular carcinoma (HCC) and breast cancer. Stimulation of Axl by the ligand Gas6 enhances tumor cell invasion and migration, and Gas6/Axl complexes enhance the expression of transcription factors that promote epithelial-mesenchymal transition (EMT) [10–12]. EMT in tumor cells is closely associated with the expression of E-cadherin (E-ca), the transcription factor Snail (SNAI1), and N-cadherin (N-ca) [13, 14]. Axl is also an important marker of EMT in ESCC cells. The Gas6/Axl axis affects various cell functions, including invasion, migration, and proliferation. However, the molecular mechanisms of Gas6-mediated cell migration have not been fully elucidated [15, 16].

Qigesan (QGS) was first described in the book Yi Xue Xin Wu written by the Chinese doctor Zhongling Cheng during the Qing Dynasty. In Traditional Chinese Medicine (TCM), QGS is mainly used to treat dysphagia, or YeGe, which includes EC [17]. Studies have shown that QGS inhibits metastasis in EC patients more effectively than other standard prescription medications [18]. Clinical observations following the treatment of many EC patients in Henan and Hebei provinces, areas of China where EC incidence is particularly high, indicate that QGS can reduce recurrence and metastasis rates in patients with radical EC after disease eradication, prolong disease-free survival (DFS), and improve quality of life [19]. Studies have confirmed that Gas6 can promote EC development, and we previously found that QGS can regulate nuclear localization and expression of NF-κB by decreasing Gas6 expression [17, 20]. We therefore hypothesized that QGS inhibits EC cell invasion and subsequent EMT and migration by inhibiting Gas6 protein functions and the Gas6/Axl pathway. In this study, we explored the effects of QGS on the Gas6/Axl signaling pathway, EMT, and mobility in EC cells.

## RESULTS

### Gas6 and Axl expression in normal and carcinoma tissues from ESCC patients

**Figure 1:**
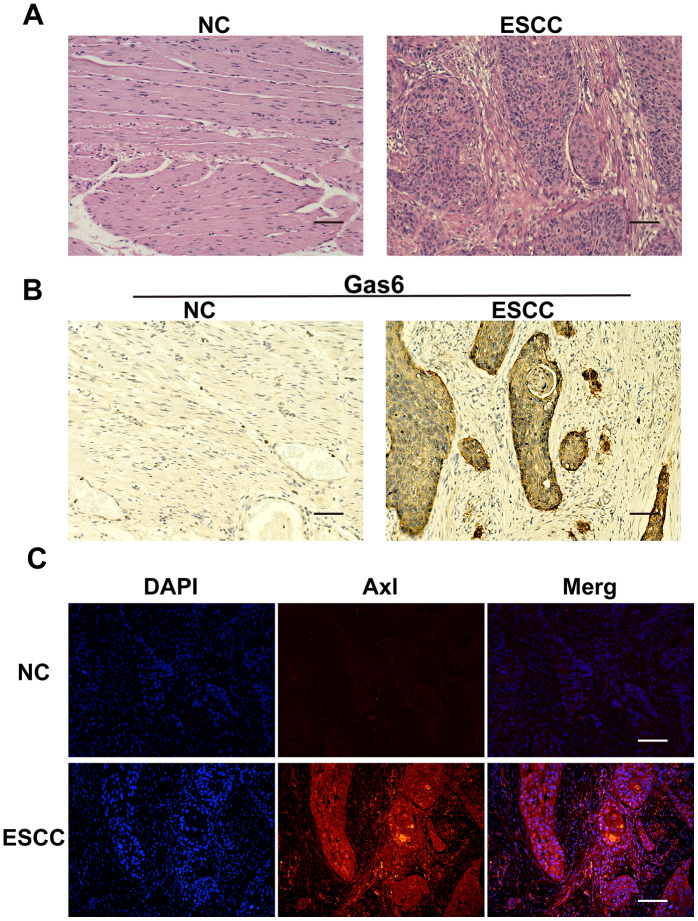
**Gas6 and Axl expression in ESCC tissues.** (**A**) HE staining; NC: nontumor esophageal tissue, ESCC: esophageal squamous cell carcinoma tissue. (**B**) Immunohistochemical assessment of Gas6 expression in NC and ESCC tissues. (**C**) Immunofluorescence assessment of Axl expression in NC and ESCC tissues; DAPI-labeled nuclei are in blue, and the merge imaged shows Axl and nuclear overlap. Scale bars indicate 50 μm.

HE staining was used to examine morphology and positioning differences between normal esophageal and ESCC tissues. Cancer cells had large, irregular nuclei and low cytoplasmic ratios (Figure 1A). Immunohistochemistry showed that Gas6 was highly expressed in ESCC carcinoma tissues, but minimally expressed in adjacent normal tissues (Figure 1B), which is consistent with findings in other tumor tissues. Similarly, immunofluorescence indicated that Axl was highly expressed in ESCC carcinoma tissues and minimally expressed in adjacent ESCC tissues (Figure 1C). These results confirmed that Gas6 and Axl are highly expressed in EC tissues.

### QGS inhibits Gas6/Axl binding

**Figure 2:**
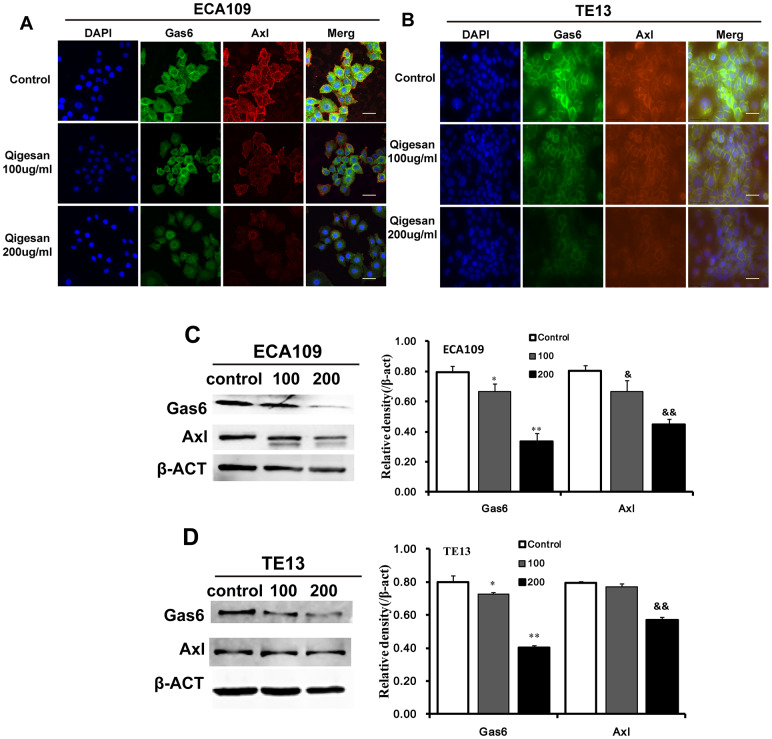
**QGS regulates the localization and expression of Gas6/Axl.** Cells were stimulated with QGS (0, 100, or 200 μg/mL) for 24 h. (**A**, **B**) Immunofluorescence images of Gas6, Axl, and Gas6/Axl channels in ECA109 and TE13 cells. Scale bars indicate 10 μm. (**C**, **D**) Western immunoblots of Gas6 and Axl. Bar graphs show significant dose-dependent inhibition of relative Gas6 and Axl protein density (normalized to β-act) after QSG stimulation compared to the unstimulated control group. Results are from three independent experiments. *p<0.05, **p<0.01, Gas6 relative protein density compared to control; ^&^p<0.05, ^&&^p<0.01, Axl relative protein density compared to control.

In order to examine the effects of QGS on cellular localization and expression of Gas6, Axl, and Gas6/Axl complexes, immunofluorescence assays were performed using the ESCC cell lines ECA109 and TE13. Gas6, Axl, and Gas6/Axl complexes were highly expressed on the cell membranes of control group cells. In comparison, cell membrane Gas6/Axl complex expression was significantly reduced in QGS-stimulated cells; Gas6 and Axl expression also decreased in these cells in a concentration-dependent manner. These effects were observed to a similar degree in both cell lines (Figure 2A, 2B). Western blotting was used to examine the effects of QGS on Gas6 and Axl protein expression. Again, QGS treatment significantly reduced the expression of Gas6 and Axl in a concentration-dependent manner, and this effect was similar in both cell types (Figure 2C, 2D).

### QGS inhibits EMT in ESCC cells

Immunofluorescence assays and confocal laser microscopy showed that E-ca was mainly expressed at relatively low levels on cell membranes and in the cytoplasm in the control group. QGS treatment increased E-ca expression on the cell membrane in a concentration-dependent manner to a similar degree in both ECA109 and TE13 cells (Figure 3A, 3D). N-ca and Snail were also mainly expressed on the cell membrane and in the cytoplasm. However, N-ca and Snail were highly expressed in the control group, and membrane expression of both gradually decreased with increasing concentrations of QGS in both ESCC cell lines (Figure 3B, 3C, 3E, 3F).

**Figure 3A-F:**
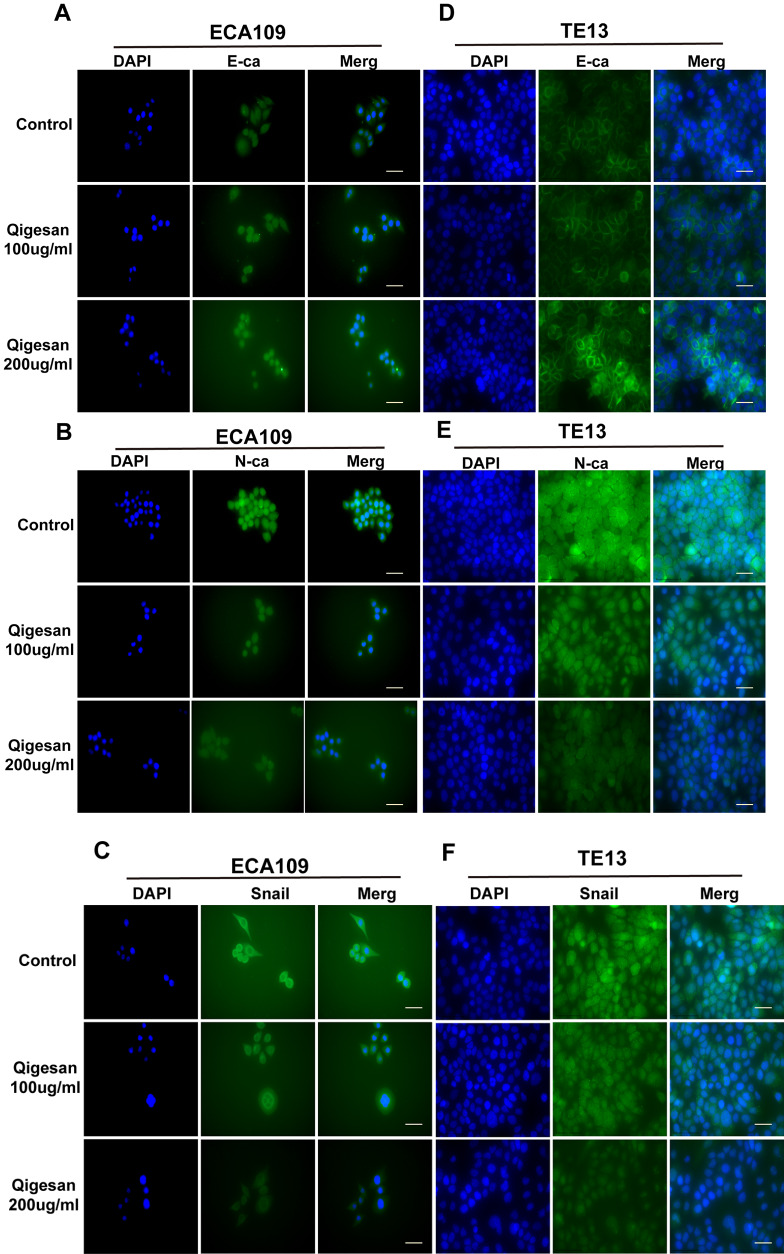
**QGS inhibits EMT in esophageal cancer cells.** (**A**–**C**) Immunofluorescence images showing E-ca, N-ca, and Snail1 localization in ECA109 cells. QSG dose-dependently increased E-ca expression and inhibited N-ca and Snail1 expression. (**D**–**F**) Immunofluorescence images of E-ca, N-ca, and Snail1 in TE13 showing the same trend after QSG stimulation. Scale bars indicate 10 μm.

Western blotting was used to examine the effects of QGS on E-ca, N-ca, and Snail protein expression. As QGS concentrations increased, E-ca protein expression increased and N-ca and Snail decreased in a dose-dependent manner. Although QGS-induced inhibition of N-ca was stronger in TE13 cells than in ECA109 cells, the direction of the effects was similar in both cell lines (Figure 3G, 3H). This indicates that QGS regulates the expression of three important EMT-associated proteins in EC.

**Figure 3G-H:**
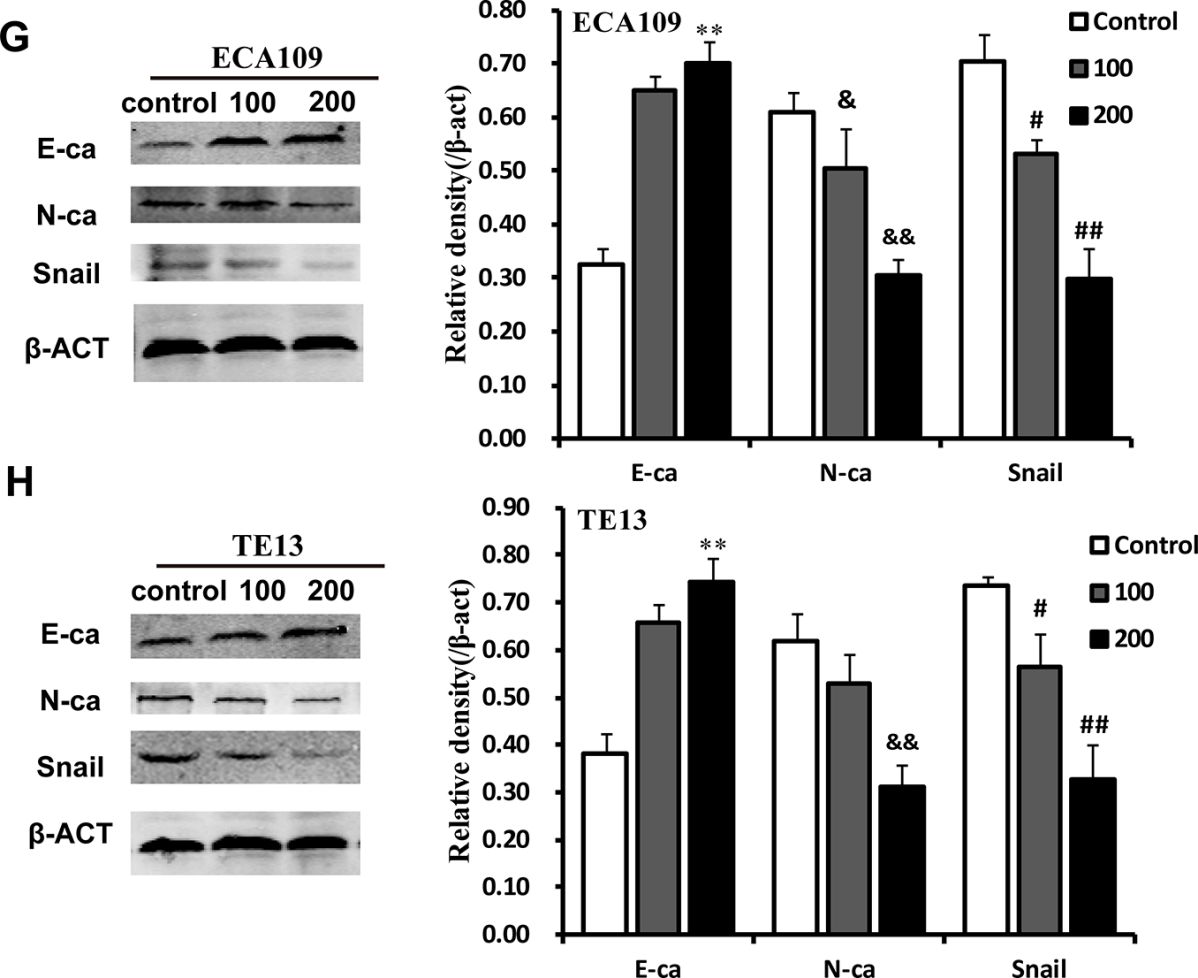
**QGS inhibits EMT in esophageal cancer cells.** (**G**, **H**) Representative Western immunoblots. Bar graphs show significant dose-dependent increases in E-ca and decreases in N-ca and Snail1 relative protein density (normalized to β-act) after QSG stimulation compared to the unstimulated control group. Results are from three independent experiments. **p<0.01, E-ca relative protein density compared to control; &p<0.05, &&p<0.01, N-ca relative protein density compared to control; #p<0.05, ##p<0.01, Snail relative protein density compared to control.

### Effects of QGS on microfilament arrangement in ESCC cells

**Figure 4:**
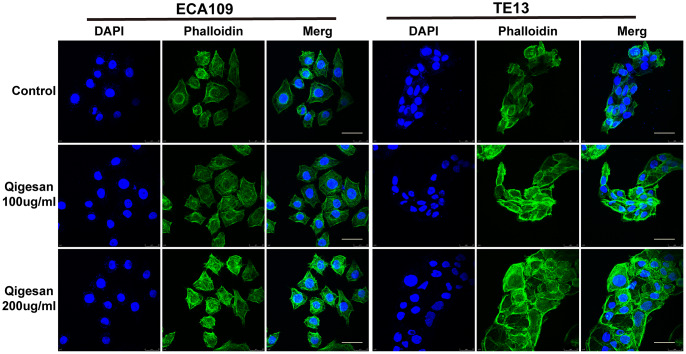
**ECA109 and TE13 cytoskeleton and microfilament structures.** After QGS stimulation, isotropic arrangement of microfilaments was enhanced in ECA109 and TE13 cells; microfilament arrangement was disordered in the control group. In addition, microfilament arrangements were more regular and numbers of pseudopods were reduced after 200 μg/mL of QGS. Scale bars indicate 10 μm.

The effects of QGS on microfilament arrangement in ESCC cells were observed using a laser confocal microscope. Microfilaments were arranged more regularly in ESCC cells stimulated with QGS; as QGS concentration increased, the cellular microfilament skeletons became more isotropic and cell morphology became increasingly regular. In contrast, microfilament arrangement was more irregular in the control group, especially in the cell membrane, where small outward-protruding pseudopods were common (Figure 4).

### QGS inhibits ESCC cell migration

**Figure 5:**
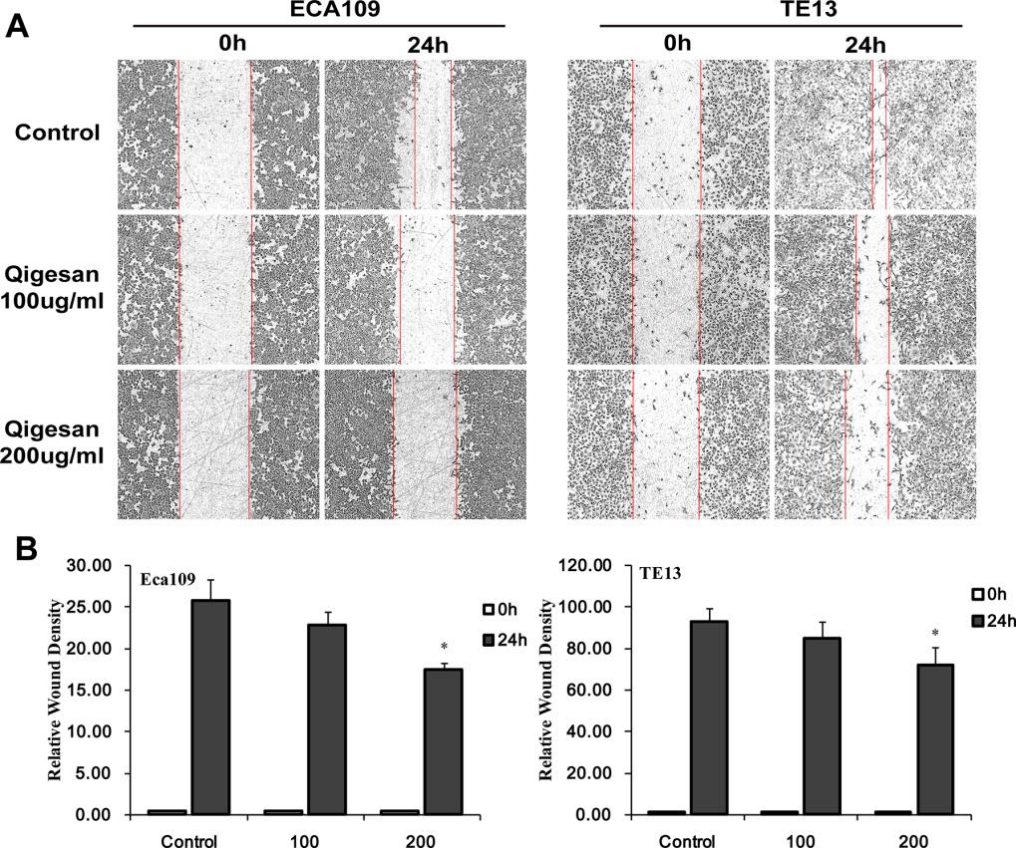
**QGS inhibits ESCC cell migration.** Scratch areas were analyzed after ECA109 and TE13 cells were stimulated with QGS (0, 100, or 200 μg/mL). (**A**) Representative photomicrographs of unstimulated and QGS-stimulated cells at 0 h and 24 h. Red lines indicate migrating edges. (**B**) Bar graphs showing the percentage relative wound density (RWD) in stimulated cells compared to controls. Results are from three independent experiments; *p<0.05.

A cell scratch test was performed to determine whether QGS inhibited migration of ECA109 and TE13 ESCC cells. In both cell lines, increasing QGS concentrations inhibited rates of cell migration into the scratch area in a time- and concentration-dependent manner; 200 μg/mL QGS resulted in significantly stronger inhibition than the 100 μg/mL dose (Figure 5). These results show that QGS can significantly inhibit the migration of ESCC cells.

### QGS inhibits invasion of ESCC cells

**Figure 6:**
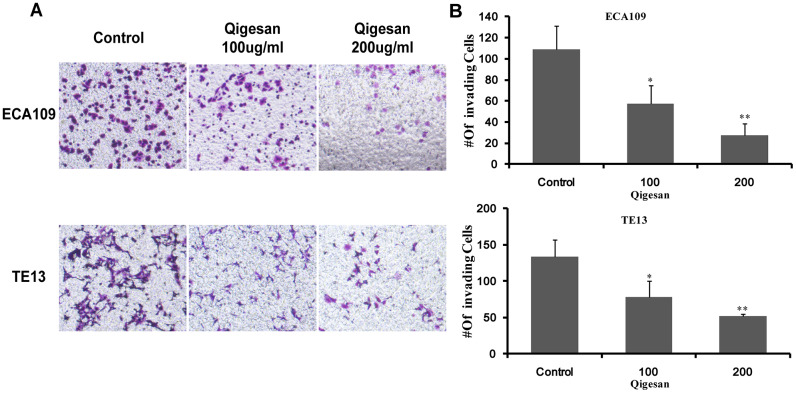
**QGS inhibits ESCC cell invasion.** (**A**) Invasion assays were performed by placing ECA109 and TE13 cells treated with QGS (0, 100, or 200 μg/mL) in the transwell system for 24 h; the control is shown on the left. (**B**) Bar graphs showing number of invading cells for each cell line after 0, 100, or 200 μg/mL QGS. Combined results from three independent experiments are shown. *p<0.05, **p<0.01, compared to control.

Next, cell invasion experiments were performed with ECA109 and TE13 cells to examine whether QGS inhibited ESCC cell invasion. Compared to the control group, the QGS groups in both cell lines exhibited a gradual concentration-dependent decrease in the number of ESCC cells passing through the transwell membranes; this effect was significantly stronger in the high-dose group (200 μg/mL) than in the low-dose group (100 μg/mL) (Figure 6). These results showed that QGS inhibited the invasive ability of ESCC cells.

## DISCUSSION

Poor tolerance of radiotherapy and chemotherapy after surgery, as well as tumor invasion and metastasis, can lead to death in EC patients. Tumor metastasis is mainly caused by tumor cell migration and invasion [25–27]. Studies have shown that Gas6 and Axl are highly expressed in gastric, ovarian, and liver cancers, among many others, and patient prognosis is negatively correlated with Gas6/Axl complex levels [10, 28, 29]. Gas6/Axl can promote bone marrow and lung cancer metastasis and invasion as well as prostate cancer cell survival [30, 31]. In this study, we found that Gas6 and Axl expression was significantly higher in pathological carcinoma tissues from human EC patients than in adjacent normal tissues. This is consistent with findings from studies of gastric cancer. ESCC cell line experiments also showed that Gas6 and Axl were highly expressed in the control group and formed Gas6/Axl complexes on the cell membrane.

According to TCM, “insufficiency of Yin” is the central cause of EC development and progression, and treatments that “nourish Yin” are recommended [17]. QGS has been used as the prototypical prescription for “nourishing Yin” in China and East Asian countries for nearly 300 years. Clinical observations indicate that QGS can alleviate airway obstruction, dysphagia, pain, nausea, vomiting, and other symptoms, reduce the toxicity and side effects of radiotherapy and chemotherapy, and inhibit recurrence and metastasis after surgery in EC patients [19]. No hepatorenal or other toxicities were observed when mice were fed QGS for 12 weeks. In addition, previous research has shown that QGS increases the distribution and expression of Cx26 and Cx43 on the cell membrane, which enhances gap junction (GJ) function and inhibits EC cell invasion and migration. Based on those results and results from our previous CCK8 experiments, QGS showed no obvious cytotoxicity at 200 μg/mL; 100 and 200 μg/mL QGS were therefore used as treatment concentrations in the current study [17, 21].

As a ligand, Gas6 activates Axl, an important member of the receptor tyrosine kinase family, triggering a series of downstream signaling pathways that promote EC cell proliferation, invasion, and migration [32, 33]. EMT is a key process in tumor cell metastasis [34, 35]. While E-ca inhibits EMT, Snail promotes EMT-induced cell migration; N-ca is highly expressed in ESCC tissues and is also related to tumor metastasis [36]. Studies show that Axl signaling directly affects mesenchymal state and promotes the development of invasive and metastatic phenotypes [37]. Axl, which is also activated by Gas6 and highly expressed in triple-negative breast cancer cells, promotes EMT [38]. Our protein chip test found that QGS can decrease Gas6 expression in EC cells. Here, we found that QGS significantly inhibits Gas6 and Axl protein expression and prevents them from binding, thereby reducing Gas6/Axl complex formation.

Studies have indicated that QGS can inhibit EC lung metastasis, which may be related to EMT, in nude mice [21]. Lung cancer cell migration is caused by Axl-mediated EMT [39, 40]. In this study, QGS stimulation enhanced E-ca expression, reduced N-ca and Snail expression, and activated cytoskeletal rearrangement and isotropic arrangement of cell microfilaments in ESCC cells. These changes occurred after QGS inhibited Gas6/Axl complex expression, and wound healing and transwell experiments showed that QGS also suppressed ESCC cell invasion and migration.

In summary, we found that QGS inhibits formation of the Gas6/Axl complex and suppresses EMT, which in turn inhibits EC cell mobility. These findings indicate that Gas6/Axl may also prove an effective target for novel EC treatments.

## MATERIALS AND METHODS

### Preparation and composition of QGS

QGS Granule Prescription was obtained from Shineway Pharmaceutical Co., Ltd. (Shijiazhuang, Hebei, China); its composition is based on the work of the famous Qing Dynasty doctor Cheng Zhongling [17, 21]. The production of all Granule Prescriptions meets GMP standards and has been approved by the Hebei Food and Drug Administration, and all botanicals have been certified as compliant with the Traditional Chinese Medicines Integrated Database (TCMID, http://119.3.41.228:8000/tcmid/search/) and The Plan List (http://www.theplantlist.org/). All Granule Prescription medicines were diluted in 100 mL of RPMI-1640 medium and filtered through a 0.22 μm syringe filter before use. The QGS concentrations used in this study were chosen based on the results of our previous experiments [17, 21].

### Gas6 and Axl expression in ESCC and adjacent tissues

Twenty ESCC patients who provided cancer and paracancerous tissue samples were randomly selected for in-patient and outpatient examination in our hospital. All tissues were confirmed by senior pathologists. Paraffin-embedded sections of selected ESCC and adjacent normal tissues were stained with HE and used for tissue and cell morphological observations. Gas6 and Axl expression were examined using immunohistochemistry. First, paraffin sections were dehydrated, antigen repair was performed, endogenous peroxidases were quenched, and serum was used for blocking. Tissues were then incubated with anti-GAS6 primary antibody (67202, Cell Signaling Technology (CST)) and secondary antibody (G23301, Servicebio), nuclei were counterstained with DAPI, and tissues were mounted on slides and dehydrated. The intelligent high-content cell imaging analysis system Invitrogen EVOS FL Auto2 (Thermo Fisher Scientific, Waltham, MA, USA) was used to observe and photograph the tissues [22]. Axl expression was detected using an immunofluorescence method [23] which involved tissue section dewaxing, antigen repair, autofluorescence quenching, incubation with primary antibody (bs-5180R, Bioss) and fluorescent secondary antibody (GB21303, Servicebio), and DAPI counterstaining of nuclei. After slides were coverslipped, EVOS FL Auto2 was used for observation and image collection.

### Reagents and antibodies

Newborn calf serum (NCS, Biological Industries, Beit Haemek, Israel), phosphate buffered saline (PBS, Biological Industries, Beit Haemek, Israel), and trypsin-EDTA digestion solution (Solarbio, Beijing, China), RPMI 1640 cell culture medium (Corning, Steuben County, New York, USA), and DAPI (D9542-1MG, Sigma Life Science, MO, USA) were used.

### Cell lines and cell culture

Human ECA109 and TE13 ESCC cell lines were obtained from Shanghai Cell Bank, Stem Cell Bank of the Chinese Academy of Sciences and Hebei Cancer Institute (Shijiazhuang, Hebei, China) and passed genotype identification. Cells were placed in RPMI-1640 culture medium containing 10% NCS and cultured in a cell incubator with 5% CO_2_ at 37°C.

### Immunofluorescence staining

ECA109 and TE13 cells were seeded on coverslips in a 24-well plate at 2 × 10^4^ and 5 × 10^4^ cells/well, respectively, treated with QGS (0, 100, or 200 μg/mL) for 24 hours, and fixed with 4% paraformaldehyde. Cell membranes were then permeabilized with 0.3% Triton X-100. After blocking with 10% BSA serum, cells were incubated with the following primary antibodies at 4°C overnight: anti-GAS6 (67020, CST), anti-mouseAXL (ab89224, Abcam), anti-E-cadherin (ET1607-75, Hangzhou HuaAn Biotechnology Co., Ltd), anti-N-Cadherin (ET1607-37, Hangzhou HuaAn Biotechnology Co., Ltd), and anti-Snail1 (ER1706-22, Hangzhou HuaAn Biotechnology Co., Ltd). Cells were then incubated with the appropriate FITC or TRITC-conjugated secondary antibody (KLP) at room temperature for 1 hour. Unbound antibody was washed away, and the cells were either mounted on glass slides with mounting medium containing DAPI or placed directly in a confocal laser scanning microscope system (Leica Microsystems, Heidelberg, Germany). Cells were observed and images captured using EVOS FL Auto2.

### Western blotting analysis

ESCC cells were seeded into cell culture flasks, and stimulated with QGS (0, 100, or 200 μg/mL) for 24 hours. Cells were then washed twice with prechilled PBS, harvested by scraping, centrifuged at 10,000 × g for 5 minutes at 4°C, and lysed on ice for half an hour with whole cell lysis buffer. The lysate was then centrifuged at 12,000 × g for 20 minutes, and the supernatant was collected and stored at -80°C for later use. Protein samples were separated using 4% - 12% NuPAGE Novex SDS gels and electrophoretically transferred to a polyvinylidene fluoride membrane (Millipore, Billerica, MA, USA). The membrane was blocked with 5% bovine serum albumin (BSA Sigma St. Louis, MO) and incubated at room temperature for 2 hours. The membranes were then incubated the with the following primary antibodies at 4°C overnight: anti-GAS6 (67202, CST), anti-rabbit AXL (8661, CST), anti-E-cadherin (ET1607-75, Hangzhou HuaAn Biotechnology Co., Ltd), anti-N-cadherin (ET1607-37, Hangzhou HuaAn Biotechnology Co., Ltd), anti-Snail1 (ER1706-22, Hangzhou HuaAn Biotechnology Co., Ltd), and anti-β-ACT (AC026, Abclonal Technology). A fluorescent dye-labeled secondary antibody was then added to the membrane and incubated at room temperature for 1.5 hours. The membrane was washed three times with Tris-buffered saline (Sigma) and Tween-20 (Sigma) for 10 minutes, and then imaged using the Odyssey infrared imaging system (LI-COR Biosciences). Protein levels were calculated as the ratio of protein band intensity to β-act intensity. Each experiment was repeated three times.

### Cytoskeleton experiment

ECA109 and TE13 ESCC cells were seeded on round glass slides in a 24-well plate at 3 × 10^4^ cells/well. After the cells had adhered, they were treated with QGS (0, 100, or 200 μg/mL) for 24 hours, washed 3 times with PBS, fixed with 4% paraformaldehyde for 20 minutes at room temperature, and washed 3 times with 1 × PBS. Phalloidin (1:1000, P5282, Sigma, USA) was then added to each well, followed by incubation at 37°C in the dark for 1 hour. Finally, the slides were coverslipped using mounting medium containing DAPI (Prolong Gold Antifade Reagent with DAPI, 8961, CST). A Leica Microsystems (Heidelberg, Germany) microscope was used to observe and take photos.

### *In vitro* cell scratch assay

ECA109 and TE13 cell migration and invasion were analyzed using IncuCyte ZOOM according to the manufacturer’s instructions (Essen Bioscience, Hertfordshire, UK). Briefly, 4 × 10^3^ ECA109 or TE13 cells/well were seeded in a 96-well culture plate at 37°C and placed in a 5% CO_2_ incubator overnight. Scratches were then made with a scratcher (Essen Bioscience), and the ESCC cells were washed twice in medium and stimulated with QGS (0, 100, or 200 μg/mL) for 24 h. The cell culture plates were placed on the IncuCyte ZOOM system and images of the scratched areas (10 × magnification) were taken at 0 and 24 h. The relative scratch density (RWD) value provided by IncuCyte software represents the rate of wound closure; an RWD value of 0 indicates that cell confluence at the wound surface is equal to cell confluence outside the wound surface. Changes in cell density tended to be consistent between experiments.

### Cell invasion assays

A transwell system (BD Biosciences, San Jose, CA, USA) was used to detect tumor cell invasiveness [24]. Cells were stimulated with QGS (0, 100, or 200 μg/mL) for 24 h. A 40 μL polycarbonate membrane was then coated with basement membrane matrix (1.5 mg/mL; BD Biosciences, San Jose, CA) at 37°C for 4 h to reconstitute the basement membrane. 100 μL of bovine serum-free cells (1 × 10^6^/mL) were added to the upper chamber of the Transwell chamber, and 0.5 mL of culture medium containing bovine serum was placed in the lower chamber. After incubation overnight at 37°C with 5% carbon dioxide, a cotton swab was used to gently scrape the cells from the upper chamber. The transwell chamber was placed in a new plate, and the upper and lower chambers with their attached contents were fixed with 4% paraformaldehyde for 20 minutes and stained with crystal violet for 30 minutes. After washing with PBS, cells were observed and photos were taken under a microscope.

### Statistical analysis

SPSS 21.0 software (SPSS, Inc. Chicago, USA) was used for statistical analysis of experimental results. Data are represented as the means ± standard deviation, and differences between groups were analyzed using One Way ANOVAs and independent sample t-tests. P < 0.05 indicated statistically significant differences.
